# Influence of positive end-expiratory pressure on four-chamber longitudinal strain analysis by speckle tracking echocardiography

**DOI:** 10.1186/cc12125

**Published:** 2013-03-19

**Authors:** F Franchi, A Faltoni, M Cameli, S Cecchini, M Lisi, M Contorni, S Mondillo, S Scolletta

**Affiliations:** 1 University of Siena, Italy

## Introduction

Speckle-tracking echocardiography (STE) has emerged as an ultrasound technique for accurately evaluating myocardial function also in critically ill patients. By tracking the displacement of the speckles during the cardiac cycle, the strain rate can be measured offline after adequate image acquisition. The aim of the study was to evaluate the effects of the positive end-expiratory pressure (PEEP) on four-chamber longitudinal strain (LS) analysis in critically ill patients.

## Methods

We enrolled 20 consecutive patients (mean age 64 ± 18) who needed mechanical ventilation and were admitted to the ICU due to heterogeneous causes. Inclusion criteria were: hypoxia requiring PEEP titration, invasive arterial pressure monitoring, age >18. Exclusion criteria were: myocardial dysfunction, cardiac arrhythmias and valvular pathologies. The same operator performed three standard echocardiography measurements (MyLab 70 Xvision; Esaote), each of them after having increased PEEP at 5, 10, and 15 cmH_2_O (T1, T2, T3, respectively). Blood gas analysis, respiratory, and hemodynamic parameters provided by a pulse contour method were also recorded. STE analysis was performed offline (XStrain™MyLab 70 Xvision; Esaote).

## Results

Left peak atrial LS (LA-PALS) was significantly reduced from T1 to T2, and from T2 to T3 (40.2 ± 12%, 35.9 ± 9%, 28.4 ± 8%, T1, T2, T3, respectively; *P <*0.05). Right peak atrial LS (RA-PALS) and right ventricular (RV)-LS showed a significant reduction only at T3 (RA-PALS: 44.7 ± 48.5% at T1, 35.9 ± 11% at T3; RV-LS: -20.2 ± 2% at T1, -16.3 ± 1.1% at T3; *P <*0.05). Left ventricular (LV)-LS did not change significantly during titration of PEEP. Cardiac chambers' volumes and cardiac output (CO) showed a significant reduction at higher levels of PEEP. Pulse pressure variation was significantly affected by higher levels of PEEP (*P <*0.05).

## Conclusion

In hypoxic patients with normal cardiac function, PEEP titration determined a reduction of LA-LS, RA-LS and RV-LS values. LV-LS values were not influenced by PEEP changes. The fall in CO, observed with higher values of PEEP, seemed to be related to the impairment of preload and not of myocardial contractility. Whenever interpreting data on cardiac function obtained with longitudinal strain analysis, attention of the clinician should be drawn to different levels of PEEP. The higher the PEEP, the more the probability of misleading interpretation of STE data.

